# FTSJ2, a Heat Shock-Inducible Mitochondrial Protein, Suppresses Cell Invasion and Migration

**DOI:** 10.1371/journal.pone.0090818

**Published:** 2014-03-04

**Authors:** Cheng-Wei Lai, Hsiao-Ling Chen, Ken-Yo Lin, Fang-Chueh Liu, Kowit-Yu Chong, Winston T. K. Cheng, Chuan-Mu Chen

**Affiliations:** 1 Department of Life Sciences, Agricultural Biotechnology Center, iEGG center, National Chung Hsing University, Taichung, Taiwan; 2 Department of Bioresources, Da-Yeh University, Changhwa, Taiwan; 3 Department of Animal Nutrition, Livestock Research Institute, Council of Agriculture, Tainan, Taiwan; 4 Department of Medical Biotechnology and Laboratory Science, Chang Gung University, Tao-Yuan, Taiwan; 5 Department of Animal Science and Biotechnology, Tunghai University, Taichung, Taiwan; Wayne State University School of Medicine, United States of America

## Abstract

Ribosomal RNA large subunit methyltransferase J (RrmJ), an *Escherichia coli* heat shock protein, is responsible for 2′-O-ribose methylation in 23S rRNA. In mammals, three close homologs of RrmJ have been identified and have been designated as FTSJ1, FTSJ2 and FTSJ3; however, little is known about these genes. In this study, we characterized the mammalian FTSJ2, which was the most related protein to RrmJ in a phylogenetic analysis that had similar amino acid sequence features and tertiary protein structures of RrmJ. FTSJ2 was first identified in this study as a nucleus encoded mitochondrial protein that preserves the heat shock protein character in mammals in which the mRNA expressions was increased in porcine lung tissues and A549 cells after heat shock treatment. In addition, a recent study in non-small cell lung cancer (NSCLC) suggested that the *FTSJ2* gene is located in a novel oncogenic locus. However, our results demonstrate that the expression of *FTSJ2* mRNA was decreased in the more invasive subline (CL1-5) of the lung adenocarcinoma cells (CL1) compared with the less invasive subline (CL1-0), and overexpression of FTSJ2 resulted in the inhibition of cell invasion and migration in the rhabdomyosarcoma cell (TE671). In conclusion, our findings indicate that mammalian FTSJ2 is a mitochondrial ortholog of *E. coli* RrmJ and conserves the heat shock protein properties. Moreover, FTSJ2 possesses suppressive effects on the invasion and migration of cancer cells.

## Introduction

Heat shock proteins (HSPs) and their biological functions are highly conserved from *Escherichia coli* to mammals [1]. The following five major classes of HSPs have been defined: HSP70s, HSP60s, HSP90s, HSP110s, and small HSPs. Protein folding, refolding and disaggregation are well-known functions of HSPs [1], [2]. However, there are still a number of proteins involved in the heat shock response that remain uncharacterized upon heat shock stress [3]. The *E. coli* enzyme RrmJ, a 23S rRNA 2′-O-ribose methyltransferase (MTase) that was identified as a novel heat shock protein, is the first gene of the *rrmJ-ftsH* heat shock operon and was first discovered in 1991 [3], [4]. RrmJ is required for the 2′-O-ribose methylation of U_2552_ in 23S rRNA [5]. Um_2552_ is one of the four 2′-O-ribose methylated nucleotides in *E. coli* rRNA and is located in the A-loop of the peptidyl transferase center of the ribosome [5], [6]. In a previous study, the *E. coli rrmJ* deletion strain lost its adaptive ability upon heat shock stress [7]. This loss may have resulted from a change in the A-loop conformation and ribosome dissociation [7]–[9].

The heat shock protein RrmJ is conserved in both *Prokaryota* and *Eukaryota* [10]. In *Saccharomyces cerevisiae*, there are three homologs of RrmJ including Trm7p, Mrm2p, and Spb1p. Trm7p is a tRNA 2′-O-ribose MTase that catalyzes the methylation of the nucleotides at positions 32 and 34 in the anticodon loop of tRNA^Phe,Trp^ [11]. Mrm2p is a mitochondrial rRNA 2′-O-ribose MTase but is encoded by the nuclear genome. Similar to RrmJ, Mrm2p catalyzes the formation of Um_2791_, which is located in the peptidyl transferase center in mitochondrial 21S rRNA in a position equivalent to that of Um_2552_ in *E. coli*. The *mrm2* deletion strain shows growth defects in a glycerol-containing medium at 37°C, indicating that this protein is important for mitochondrial function and is crucial for heat shock adaptation in yeast [12]. Spb1p is a nuclear protein composed of 841 amino acids and contains two functional domains: a small, N-terminal, RrmJ-like domain and a large, C-terminal domain that is involved in rRNA maturation. Spb1p catalyzes the methylation of U_2921_ and G_2922_ in the A-loop of 25S rRNA, which are equivalent to U_2552_ and G_2553_ in *E. coli* 23S rRNA, respectively [13]–[16]. *E. coli* RrmJ and its three homologs in yeast all contain the four-residue sequence, K-D-K-E, which composes the catalytic center of RrmJ [10], [17]. Moreover, the structures of the substrates of these three homologs and the positions of the methylated rRNA residues are very similar to those of *E. coli* RrmJ [6], especially Mrm2p, because it is located in the mitochondria, which are believed to be degenerated bacteria [18].

In mammals, there are also three RrmJ homologs, designated as FTSJ1, FTSJ2, and FTSJ3, that correspond to Trm7p, Mrm2p, and Spb1p, respectively. In previous studies, the mutation of human *FTSJ1* has been shown to cause nonsyndromic X-linked mental retardation (NSXLMR) in Japanese families [19]. A *FTSJ2* locus in the genome, gene amplification and mRNA over-expression were discovered in several non-small cell lung cancer (NSCLC) tissue samples [20], and FTSJ3 was revealed to function in pre-rRNA processing [21], [22]. However, little is known about these three homologs in mammals. Thus, in this study, we used *E. coli* RrmJ as a starting point to construct a phylogenetic tree containing several typological species and mammals, which showed that FTSJ2 is an ortholog of RrmJ. Based on the highly conserved FTSJ2 protein sequences within mammals, we established the basic characteristics of FTSJ2 and its gene expression during the heat shock response in different porcine tissues and human cancer cells. Because previous studies have shown the abnormal expression of FTSJ2 in NSCLC, we further investigated the functions of FTSJ2 in cell invasion and migration using human lung adenocarcinoma and rhabdomyosarcoma cell lines.

## Materials and Methods

### Phylogenetic Analysis of the *E. coli* RrmJ Homologs

The RrmJ domain of 39 protein sequences and the three out-group proteins, fibrillarin (PDB code: 1FBN) [10], [23], vaccinia VP39 (1AV6) [24] and catechol-O- methyltransferase (1VID) [25], which are structurally and functionally similar to *E. coli* RrmJ, were used for the construction of a phylogenetic tree. The distance matrix was calculated using the JTT model. The minimum evolution (ME) method with 1,000 bootstrap replicates was performed using the MEGA5 program (www.megasoftware.net/) [26]. The nodes of the tree with a bootstrapping support of >50% are shown.

### Database Search for RrmJ Homologs and the Multiple Sequence Alignment

BLASTp was used to search the complete protein sequences in the non-redundant (nr) database at the National Center for Biotechnology Information (NCBI) website. Fourteen proteins from humans, *Methanococcus jannaschii* and three invertebrate species were obtained using *E. coli* RrmJ as a query, and an E-value of <3e-08 was defined as the cut-off value. Twenty-two vertebrate proteins were found using human FTSJ1, FTSJ2 and FTSJ3 as the queries, and a 50% amino acid identity was defined as the cut-off value in this search. The putative RrmJ domains of the 38 protein sequences were aligned with that of *E. coli* RrmJ using ClustalW and were slightly adjusted according to their predicted secondary structures, which were calculated using the PORTER query (distill.ucd.ie/porter/) [27].

### The Animals and the Heat Stress Treatment

Twelve three-month-old female Landrace×Yorkshire crossbred (LYC) piglets were purchased from the Animal Industry Division of the Livestock Research Institute of the Council of Agriculture (COA) (Tainan, Taiwan). The procedures used in this study were approved by the Institutional Animal Care and Use Committee (IACUC) of the COA Livestock Research Institute (Approval No. 98021). The piglets (n = 4) were raised at 25°C and 60% humidity in animal houses, which were equipped for temperature and humidity control [28], [29]. For the heat stress treatment, the piglets that were raised at room temperature (25°C) were exposed to heat shock temperatures of 30°C or 35°C and maintained at 60% humidity for 1 week. The piglets were then sacrificed, and tissue samples from 11 organs were isolated for total RNA extraction.

### Cell Culture

The cancer cell lines of HepaG2 (ATCC No. HB-8065), TE671 (ATCC No. CCL-136), and A549 (ATCC No. CCL-185) were purchased from American Type Culture Collection (ATCC; Manassas, VA, USA). The lung adenocarcinoma CL1 sublines CL1-0 and CL1-5 were kindly provided by Dr. Jeremy J.W. Chen, National Chung Hsing University, Taichung, Taiwan [30]. All of the cell lines were grown in Dulbecco’s Modified Eagle’s Medium (DMEM; Invitrogen Corp., Grand Island, NY, USA) containing high glucose (4500 mg/L) and supplemented with 10% fetal bovine serum (FBS) at 37°C and 5% CO_2_. At 80% confluence, the cells were subcultured at a ratio of 1∶3 to 1∶5, and the medium was changed every three days as described previously [31], [32].

### Heat Shock Treatment of the Cells

In our heat shock response analysis, the cancer cells that grew at 37°C and 5% CO_2_ were subjected to heat shock at 42°C or 45°C and 5% CO_2_ for 1 hour. After this heat shock treatment, the cells were transferred to 37°C for 0, 3 or 6 hours and then harvested for total RNA extraction.

### Cell Transfection via Electroporation

The 6.74-kb pCMV-h*FTSJ2*-IRES2-*DsRed* plasmid was constructed by inserting the full-length human *FTSJ2* (h*FTSJ2*) protein coding sequence into the *Eco*RI restriction site of the pIRES2-*DsRed2* vector (Clontech Laboratories Inc., Mountain View, CA, USA). The TE671 and HepG2 cell lines were transfected with this plasmid via electroporation with a BTX ECM2001 system (BTX, Holliston, MA, USA). Briefly, 6×10^6^ TE671 or 2×10^7^ HepG2 cells were suspended in 400 µL of DMEM, which contained 5 µg or 50 µg of plasmid DNA, respectively, and then, the cells were subjected to electroporation at 200 V for 4 msec or 100 V for 30 msec, respectively. After electroporation, the cells were grown in a culture medium containing 400 µg/mL of the antibiotic G418 for the selection of cells that were stably expressing h*FTSJ2*.

### Isolation of the Mitochondrial and Cytosolic Protein Fractions

The mitochondrial and cytosolic proteins of the TE671 cell fractions were isolated using the reagent-based method of the Mitochondria Isolation Kit (Pierce, Rockford, IL, USA) according to the manufacturer’s instructions.

### Western Blot Analysis

To analyze the expression of h*FTSJ2* stable expression colonies of the TE671-h*FTSJ2* and HepG2-h*FTSJ2* cells were collected, homogenized in 300 µL of RIPA buffer (5 mM Tris-HCl [pH 7.4], 0.15 M NaCl, 1% NP-40, 0.25% sodium deoxycholate, 5 mM EDTA [pH 8.0] and 1 mM EGTA), held on ice for 30 min and then centrifuged at 14,000 rpm for 30 min. The supernatants were collected as the total protein lysate. The supernatants (20 µg) were then separated by SDS-PAGE in a 12% acrylamide gel (acryl:bis of 30∶0.8) and transferred to a polyvinylidene difluoride (PVDF) membrane [33], [34]. The membrane was blocked with 5% BSA (filtered through a 0.22-µm membrane) and immunoblotted with anti-hFTSJ2 (1∶1,000) and anti-GAPDH (1∶500) antibodies overnight at 4°C. After washing with phosphate-buffered saline containing Tween 20 (PBST), the membrane was incubated with the appropriate horseradish peroxidase-conjugated secondary antibody for 1 hour at 25°C, and the protein bands were detected by enhanced chemiluminescence (PerkinElmer, Waltham, MA, USA) and an ImageQuant LAS 4000 mini system (GE Healthcare Biosciences, Pittsburgh, PA, USA).

For immunoblotting of the mitochondrial and cytosolic protein fractions, 5 µg of protein from each fraction was separated by SDS-PAGE and immunoblotted with anti-hFTSJ2 (1∶1,000), anti-VDAC (mitochondrial fraction control, 1∶1,000) and/or anti-MEK-1 (cytosolic fraction control, 1∶1,000) antibodies.

### Immunofluorescence Microscopy

The TE671-h*FTSJ2* and HepG2-h*FTSJ2* cells were grown to 80% confluence in 24-well dishes. Then, the cells were fixed with 4% paraformaldehyde for 20 min and permeabilized with 0.1–0.3% Triton X-100 for 5 min, followed by three washes with PBS. The cells were blocked with horse serum for 1 hour and incubated with an anti-hFTSJ2 antibody (1∶1,000) overnight at 4°C and then with the appropriate fluorescein isothiocyanate (FITC)-conjugated antibody for 1 hour. The cells were counter-stained with MitoTracker Red CMXRos (Invitrogen Corp., Grand Island, NY, USA) to stain the mitochondria and DAPI to stain the nuclei and then mounted with glycerol. The cells were examined by laser scanning confocal fluorescence microscopy in which FITC was excited at 488 nm, MitoTracker Red was excited at 580 nm and DAPI was excited at 358 nm.

### Real-time RT-PCR and Semi-quantitative RT-PCR

The total RNA was isolated from the cell lines or the porcine tissues using the TRIzol reagent (Invitrogen Corp., Grand Island, NY, USA), according to the manufacturer’s instructions. The total RNA was treated with DNase I (1 µL of DNase I per 1 µg of total RNA) at 37°C for 30 min to eliminate any genomic DNA contamination. Then, 1 µL of a stop solution was added, and the sample was incubated at 65°C for 10 min to inactivate the DNase I. The DNase I-treated RNA (1 µg) was used directly as a template for first-strand cDNA synthesis with the ImProm-II Reverse Transcription System (Promega, Madison, WI, USA). Real-time RT-PCR was performed using the relative standard curve method with SYBR Green I (Sigma, St. Louis, MO, USA) as the dsDNA fluorescence dye. The reactions were performed using a Roter-Gene 6000 system (Corbett Life Science, Mortlake, Australia) under the following conditions: 94°C for 5 min; 40 cycles of 95°C for 30 sec, 59°C or 68°C for 30 sec and 72°C for 20 sec; and a melting curve from 65°C to 95°C [35]. Semi-quantitative RT-PCR reactions were performed under the following conditions: 94°C for 5 min and then 25 or 30 cycles at 95°C for 30 sec, 59°C or 68°C for 30 sec, 72°C for 20 sec and 72°C for 7 min. The amplified products were separated on a 1.5% agarose gel and then quantified using Kodak 1D software (Kodak, New Haven, CT, USA). The primers, which were used in real-time RT-PCR or semi-quantitative RT-PCR, are listed in Table 1.

**Table 1:** Primer sequences for real-time and semi-quantitative RT-PCR.

Species	Gene	Sense (5′-3′)	Anti-sense (5′-3′)
Human	*FTSJ2*	GCTGGTGTGTGTTTCCTTTCA	CAGAATCTGGTGCCTCTCGT
	*HSP70.2*	GCACGTTCGACGTGTCCAT	GCTTGTTCTGGCTGATGTCCTT
	*β-actin*	CCGTCTTCCCCTCCATCGTGGG	CGCAGCTCATTGTAGAAGGTGTGG
	*GAPDH*	GAGAAACCTGCCAAGTATGATG	ACCTGGTCCTCAGTGTAGCC
Pig	*Ftsj2*	ACGAGTTCCCAGGAGAATCAGA	TGCTTTGGCAACGACCTTTAA
	*Hsp70.2*	GCACGTTCGACGTGTCCAT	GCTTGTTCTGGCTGATGTCCTT
	*β-actin*	CATCACCATCGGCAACGA	TTCCTGATGTCCACGTCGC

### Wound Healing Assay

The TE671 or TE671-h*FTSJ2* cells were grown in 60-mm dishes to 90% confluence. Then, a wound was produced by scraping the cell monolayer with a sterile P200 pipette tip. During the wound healing process, time-lapse images were obtained every 10 min for 12 hours, and the cell migration area was calculated ([healing area/wounding area]×100%) using the ImageJ program (http://rsbweb.nih.gov/ij/index.html).

### Invasion Assay

The invasion assay was performed on the TE671 and TE671-h*FTSJ2* cells using Trans-well chambers with an 8-µm pore size (Merck Millipore, Darmstadt, Germany). The upper chamber of the Trans-well was pre-coated with Geltrex matrix gel (30 µL/well) (Invitrogen Corp., Grand Island, NY, USA) and inserted into the lower chamber, which contained DMEM with 10% FBS. The cells (1×10^4^) were suspended in 200 µL of DMEM and added to the upper chamber. After 12 hours, the cells on the upper surface of the membrane were removed with a cotton swab, and the cells on the lower surface of the membrane were fixed with methanol for 10 min and subjected to Giemsa staining. The Giemsa-positive area of the membrane was calculated ([Giemsa positive area/total area]×100%) using the ImageJ program.

### Statistical Analysis

All of the data are presented as the means±standard error (SE). The statistical comparisons were performed using Student’s t-test. The Duncan method of one-way ANOVA was used for multiple group comparisons. The *P*<0.05 or *P*<0.01 level was considered significant.

## Results

### FTSJ2 is Closely Related to *E. coli* RrmJ

**Figure 1:**
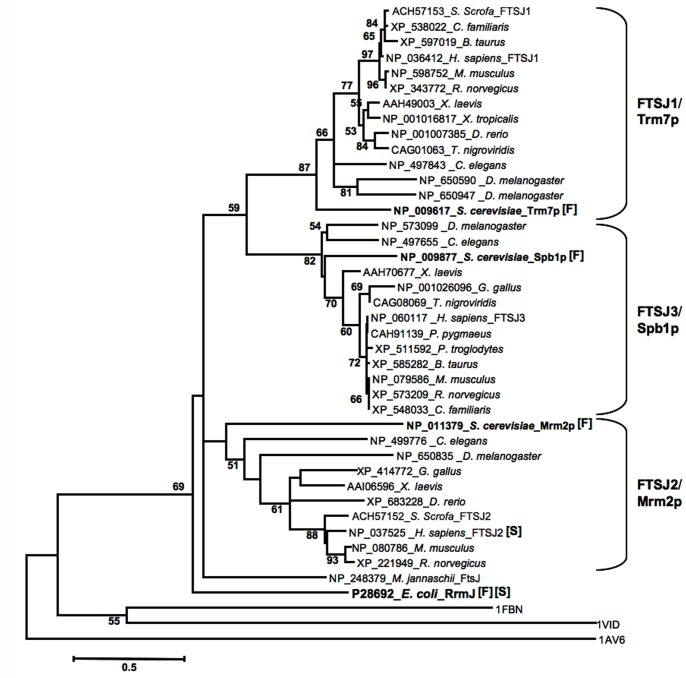
Phylogenetic tree of the *E. coli* RrmJ homologs from *Eubacteria* to *Mammalia*. This phylogenetic analysis represents the following three major homolog protein lineages: FTSJ1/Trm7p, FTSJ2/Mrm2p, and FTSJ3/Spb1p. The out-groups of the tree were catechol-O- methyltransferase, VP39, and fibrillarin (PDB code 1VID, 1AV6, and 1FBN, respectively). These out-groups were chosen because of their similar functions and structures to *E. coli* RrmJ. The number at each branch node indicates the reliability of the splits (%) through the processing of 1,000 bootstrap replicates. The GenBank accession numbers and species are shown for each protein. [F] and [S] denote proteins with known functions and protein structures, respectively.

RrmJ is a 23S rRNA 2′-O-ribose MTase and is conserved in nearly all of the different species. To evaluate the relationship among the RrmJ homologs, a phylogenic tree with 39 RrmJ homologs was constructed. The NCBI BLASTp program was used to identify the homologs of the *E. coli* RrmJ protein in *M. jannaschii*, *S. cerevisiae*, *Caenorhabditis elegans*, *Drosophila melanogaster,* and in vertebrates. Using the minimum evolution method, the phylogenic tree showed the following three phylogenies: FTSJ1/Trm7p, FTSJ2/Mrm2p, and FTSJ3/Spb1p (Figure 1). Using the JTT model to calculate the protein distances and comparing these distances within each phylogeny, the protein distances within the FTSJ2/Mrm2p group were found to be larger than those within the FTSJ1/Trm7p and FTSJ3/Spb1p groups, indicating that the RrmJ homologs FTSJ1 and FTSJ3 are more conserved between species than FTSJ2. However, the distance between *E. coli* RrmJ and the root of the FTSJ2/Mrm2p group (distance: 0.84) was shorter than the roots of the FTSJ1/Trm7p and FTSJ3/Spb1p groups (distance: 1.25 and 1.28), suggesting that the FTSJ2/Mrm2p proteins are the most related to *E. coli* RrmJ and are presumed to be the orthologs of RrmJ. This assumption is supported by the known function of *S. cerevisiae* Mrm2p. Mrm2p catalyzes the methylation of 2′-O-ribose in the mitochondrial 21S rRNA [12], which has the same rRNA structure as the substrate of RrmJ.

### The Protein Structures of the FTSJ2 Orthologs are Similar to that of *E. coli* RrmJ

**Figure 2:**
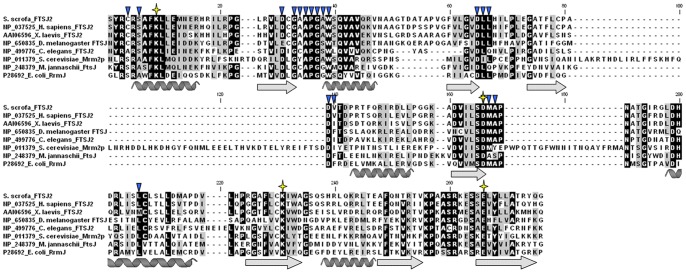
Protein sequence alignment of *E. coli* RrmJ with its FTSJ2 orthologs in 7 different species. The α-helices and β-strands were based on the RrmJ protein structure (PDB code: 1EIZ). The stars and triangles indicate the K-D-K-E catalytic center and the SAM binding residues in RrmJ, respectively. The residues with identical and similar chemical properties are highlighted in black and gray, respectively.

The protein sequence alignment of *E. coli* RrmJ, *M. jannaschii* FtsJ, *S. cerevisiae* Mrm2p, and other FTSJ2 orthologs showed many similarities. The K_50_, D_124_, K_164_ and E_199_ residues, which compose the catalytic center of RrmJ, were present in all of the RrmJ orthologs. Furthermore, the 19 residues involved in S-adenosylmethionine (S-AdoMet, SAM) binding in RrmJ were also highly conserved in FTSJ2 (Figure 2). A comparison of the similarity of the full-length amino acid sequences of human FTSJ2 with *E. coli* RrmJ, *S. cerevisiae* Mrm2p, and porcine FTSJ2, which represented the *Eubacteria*, *Eukaryota*, and *Mammalia* homologs, respectively, showed that the E-values (and amino acid identities) were 3e-43 (35%), 3e-33 (29%), and 7e-140 (76%), respectively.

In addition, a comparison of the two published three-dimensional protein structures of *E. coli* RrmJ and human FTSJ2 and a predicted structure of porcine FTSJ2 showed that these structures contain five α-helixes and seven β-strands that compose an open-sheet structure. The positions of the SAM binding residues and the amino acids of the catalytic center showed similar arrangements in the protein structures. Notably, the first residue of the catalytic center, lysine (K_50_ in RrmJ), showed an approximately 120° rotation in human FTSJ2 (PDB code: 2NYU) [36] compared with *E. coli* RrmJ (Figure S1). This rotation may account for the different structures of the rRNA substrates of the *E. coli* and the human proteins.

### FTSJ2 is Localized in the Mitochondria of Human Cells

**Figure 3:**
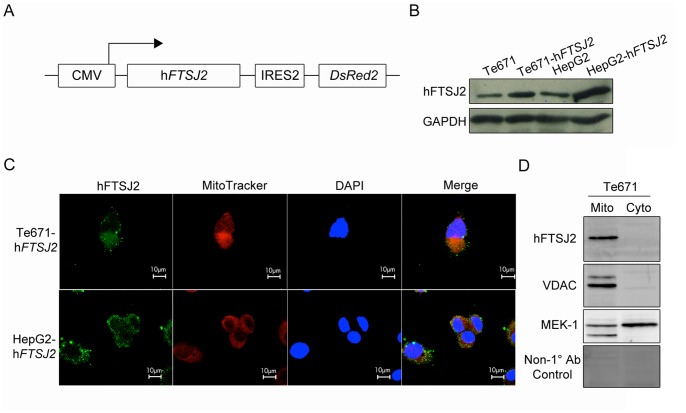
Subcellular localization of human FTSJ2 in TE671 and HepG2 cells. (**A**) Schematic of the hFTSJ2 over-expression vector. (**B**) Over-expression of the hFTSJ2 protein in the TE671-h*FTSJ2* and HepG2-h*FTSJ2* stable clones. (**C**) Immunofluorescence staining with anti-hFTSJ2 (green), MitoTracker for mitochondria (red), and DAPI for nuclei (blue) in the TE671-h*FTSJ2* and HepG2-h*FTSJ2* cells. (**D**) Mitochondrial localization of the hFTSJ2 protein in non-transfected TE671 cells. VDAC and MEK-1 were used as the mitochondrial and cytosolic fraction controls, respectively, in the Western blot analysis.

Mrm2p, the FTSJ2 ortholog in *S. cerevisiae*, is located in the mitochondria and is responsible for the 2′-O-ribose methylation of the 21S rRNA. Thus, we detected the subcellular localization of the FTSJ2 protein in the human cell lines. A vector for the overexpression of h*FTSJ2* driven by the CMV promoter was constructed (pCMV-h*FTSJ2*-IRES2-*DsRed2*; Figure 3A). The rhabdomyosarcoma (TE671) and hepatocarcinoma (HepG2) cell lines were transfected with the pCMV-h*FTSJ2*-IRES2-*DsRed2* vector, and the expression of the hFTSJ2 protein was detected by Western blot analysis. The results showed that the TE671-h*FTSJ2* and HepG2-h*FTSJ2* cells had higher expression levels of hFTSJ2 than the non-transfected cells (Figure 3B). Immunofluorescence showed that most of the hFTSJ2 protein was located in the cytoplasm but not in the nuclei in both the TE671-h*FTSJ2* and HepG2-h*FTSJ2* cells, and the mitochondria, which were stained with MitoTracker Red, showed the same localization as hFTSJ2 (Figure 3C). In the analysis of the mitochondrial and cytosolic protein fractions, hFTSJ2 was detected in the mitochondrial protein fraction but not in the cytosolic fraction (Figure 3D). These results indicated that human FTSJ2, as its ortholog in *S. cerevisiae*, is a mitochondrial protein.

### *Ftsj2* mRNA is Expressed in all of the Porcine Tissues but at Different Levels

Because of the higher similarity between human and porcine FTSJ2 (amino acid identity = 76% and RrmJ domain identity = 81%; Figure 2) and the similar physiological responses of humans and pigs, we further analyzed the expression of the porcine *Ftsj2* gene, and sequenced the coding region of the porcine *Ftsj2* mRNA (GenBank accession number: EU694400). Then, 13 tissues from a female piglet were analyzed for the expression of porcine *Ftsj2* mRNA by RT-PCR. The results showed that all of the 13 tissues expressed *Ftsj2* mRNA at different levels. The heart and kidney showed the highest expression; the large intestine, muscle, lung, spleen and liver showed mid-level expression; and the small intestine, ovary, brain, stomach, mammary gland and bladder showed the lowest expression (Figure S2).

### The Level of *Ftsj2* mRNA Expression Increases in Several Tissues after Heat Shock Stress

**Figure 4:**
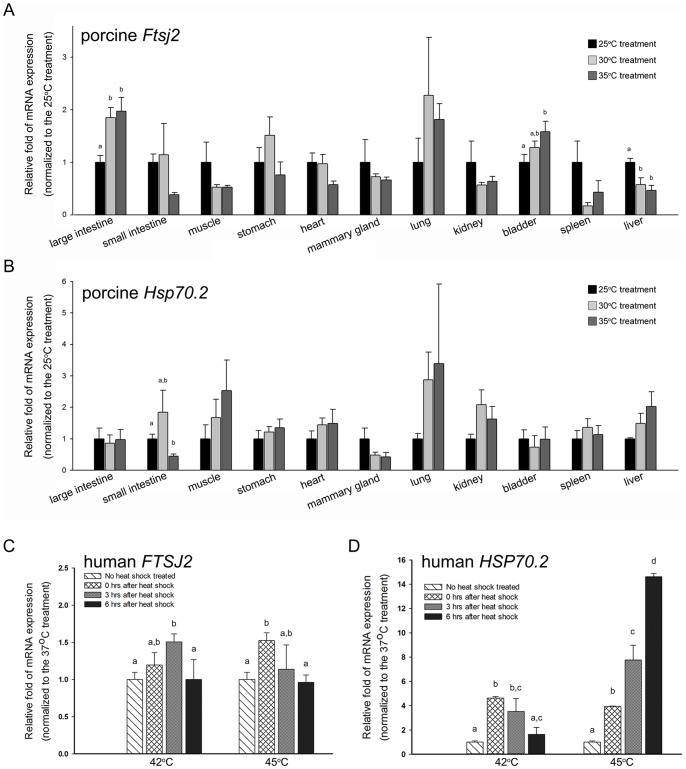
*FTSJ2* mRNA expression after *in vivo* and *in vitro* heat shock treatments. Porcine *Ftsj2* (**A**) and *Hsp70.2* (**B**) mRNA expressions in piglets, which were raised at 25°C, 30°C or 35°C for 1 week. Human *FTSJ2* (**C**) and *HSP70.2* (**D**) mRNA expressions in A549 lung cells after 1 hour of heat shock at 42°C or 45°C followed by 0, 3 or 6 hours of recovery at 37°C. Porcine *Hsp70.2* or human *HSP70.2* mRNA expression was used as the heat shock response control. The values are equal to = the means±SE; n = 4. The bars with different letters represent a significant difference (*P*<0.05).

Previous studies have shown that the mRNA of *E. coli rrmJ* increased nearly 20-folds under heat shock stress [3] and was involved in the thermal adaptation of *E. coli* [7]. Thus, the heat shock protein characteristics of mammalian FTSJ2 were also evaluated in piglets, which are known to be thermally sensitive in the livestock industry. Three-month-old female piglets were raised at room temperature (25°C), and to produce enough heat shock stress in the warm-blooded piglets, temperatures of 30°C or 35°C were used to stimulate the heat shock response for at least 1 week. The porcine *Ftsj2* mRNA expression in 11 tissues from these piglets was detected, and *Hsp70.2* mRNA expression was used as the heat shock response positive control (Figures 4A and 4B). The results showed that *Ftsj2* mRNA expression was up-regulated in the large intestine, stomach, lung and bladder and down-regulated in the small intestine, muscle, heart, mammary gland, kidney, spleen and liver in the 30°C and 35°C environments (Figure 4A). Notably, the only tissue that showed an up-regulation of both *Ftsj2* mRNA expression and *Hsp70.2* mRNA expression, which corresponds to a positive heat shock response, was the lung (Figure 4B). This result may have been caused by direct exposure of the lung tissue to the increased temperature through inhalation of the hot air.

### The Level of Human *FTSJ2* mRNA Expression Increases after Heat Shock in A549 Cells

Our results showed that *Ftsj2* mRNA was overexpressed in the porcine lung after heat shock; therefore, a human lung adenocarcinoma epithelial cell line (A549) was used to further confirm the heat shock response *in vitro* to eliminate the systematic effects observed in the piglets. A549 cells were first grown at 37°C and 5% CO_2_. For the heat shock treatment, the cells were grown at 42°C or 45°C, 5% CO_2_ for 1 hour and then returned to 37°C for 0, 3, or 6 hours, respectively. Human *FTSJ2* mRNA was detected by real-time RT-PCR. The results revealed that after both the 42°C and 45°C heat shock treatments, the h*FTSJ2* mRNA expression increased by more than 50% (50.6% and 52.6% at 3 hours and 0 hours, respectively) (*P*<0.05) (Figure 4C) compared with the non-heat shock control. The up-regulation of the *HSP70.2* mRNA indicated a positive heat shock response after the 42°C and 45°C treatments in the A549 cells (Figure 4D).

### FTSJ2 Inhibits Cancer Cell Migration and Invasion

**Figure 5:**
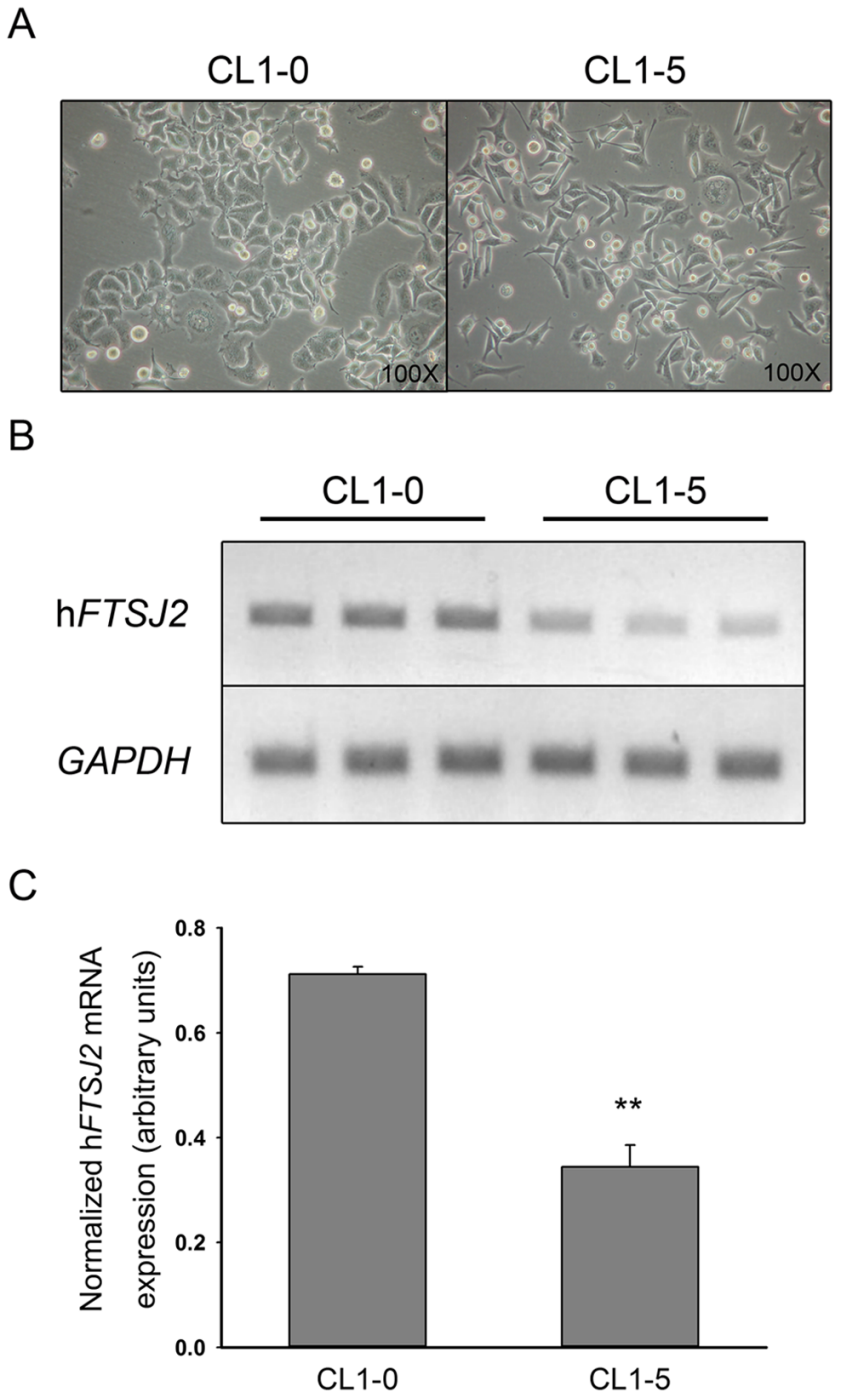
Comparison of the h*FTSJ2* mRNA expression levels in two lung cancer sublines (CL1-0 and CL1-5). (**A**) Morphology of CL1-0 and CL1-5 cells. (**B**) Determination of the h*FTSJ2* mRNA expression levels in the CL1-0 and CL1-5 cells in triplicate. (**C**) Relative quantification of the h*FTSJ2* mRNA expression. *GAPDH* mRNA was used as an internal control. The values are equal to = the means±SE; n = 3; **P<0.01 vs. the non-heat shock group.

In a recent study in clinical samples of NSCLC, the human *FTSJ2* gene was located in a novel oncogenic locus of NSCLC. These results indicate that FTSJ2 may also be involved in the growth of cancer cells. To evaluate the roles of FTSJ2 in cancer, the gene expressions in the NSCLC cell lines were detected. A human lung adenocarcinoma cell line (CL1), which was isolated from an adenocarcinoma from the lung of a 64-year-old man, has been cloned and passed on for more than 60 generations. Using the Transwell invasion chamber, the CL1 cell line was separated into six sublines according to their metastatic ability (CL1-0 to CL1-5, from lowest to highest invasiveness) [41]. The h*FTSJ2* mRNA expression was detected in two of these sublines (CL1-0 and CL1-5). Surprisingly, the more invasive CL1-5 cells showed a 50% decrease in the h*FTSJ2* mRNA expression than the less invasive CL1-0 cells (Figures 5B and 5C).

**Figure 6:**
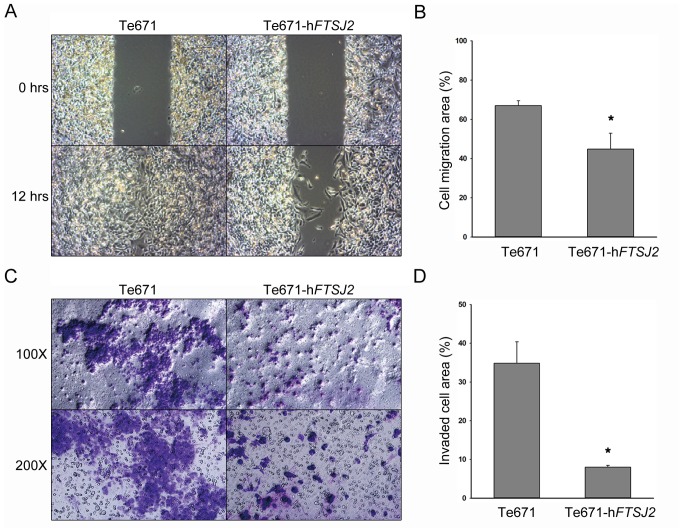
Inhibition of cell migration and invasion upon the over-expression of hFTSJ2 in TE671 cancer cells. (**A**) The wound healing assay showing that the TE671-h*FTSJ2* cells had a reduced migration compared with the untransfected TE671 cells at 12 hours after wounding. (**B**) Cell migration area at 12 hours after wounding ([healing area/wounding area]×100%). (**C**) Invasion assay showing that the TE671-h*FTSJ2* cells had a reduced invasion compared with the untransfected TE671 cells. The cells that penetrated the Trans-well membrane are shown in purple. (**D**) Quantity of the cells that invaded the Trans-well membrane ([Giemsa positive area/total area]×100%). The values are equal to = the means±SE; n = 3; *P<0.05 vs. untransfected TE671 cells.

In addition to the down-regulation, FTSJ2 increased cell invasion in the CL1-5 cells. To further evaluate the abilities of FTSJ2 to influence cell migration and metastasis, the h*FTSJ2*-overexpressed cell line (TE671-h*FTSJ2*) previously mentioned was used in the cell migration and invasion assay and was compared with the non-transfected TE671 cells. The results of our wound healing assay showed that at 12 hours after wounding, the migration area of the TE671-h*FTSJ2* cells was significantly decreased (*P*<0.01) compared with the non-transfected TE671 cells (Figures 6A and 6B). The same results were also observed in our invasion assay in which the quantity of invaded TE671-h*FTSJ2* cells per Trans-well was significantly lower than that of the non-transfected TE671 cells (Figures 6C and 6D). These results indicate that FTSJ2 is involved in the inhibition of cancer cell migration and invasion.

## Discussion

In this study, we characterized the mammalian FTSJ2 protein, which we presumed to be an ortholog of *E. coli* RrmJ. RrmJ is known as a 2′-O-ribose MTase, which methylates U_2552_ in the A-loop of the peptidyl transferase center in the 23S rRNA [5]. Um_2552_ is one of the four 2′-O-methylated nucleotides in rRNA [6]. In a previous study, a lack of U_2552_ methylation has been found to influence the tertiary interactions of U_2552_, U_2555_, and C_2556_; to reduce the conformational dynamics of the A-loop [8]; and to subsequently decrease the ribosome stability and translation efficiency [7], [9], [37]. These results indicate the importance of RrmJ and the methylation of U_2552_. In our phylogenetic analysis in this study, the RrmJ homologs clustered into the following three groups in *Eukaryota*: FTSJ1/Trm7p, FTSJ2/Mrm2p, and FTSJ3/Spb1p (Figure 1). In a comparison of the divergent distance between RrmJ and the ancestral roots of each group, FTSJ2/Mrm2p showed the closest relation to RrmJ. In the FTSJ2/Mrm2p protein group, *S. cerevisiae* Mrm2p has been studied extensively. In the mitochondrial rRNA of *S. cerevisiae*, only three nucleotides are modified, including the U_2791_ of the 21S rRNA, which is 2′-O-ribose-methylated by Mrm2p. It has been proposed that Mrm2p is the mitochondrial RrmJ ortholog in *S. cerevisiae* according to the equivalent catalytic positions of Um_2791_ in the *S. cerevisiae* mitochondrial 21S rRNA and Um_2552_ in the *E. coli* 23S rRNA [6], [12]. However, mammalian FTSJ2 remains uncharacterized. Thus, we performed a sequence alignment of human FTSJ2 with RrmJ, Mrm2p and FtsJ2 in *M. jannaschii* and three typological invertebrate species (Figure 2), and we compared the 3D structures of RrmJ, human FTSJ2 and porcine FTSJ2 (Figure S1). A previous study showed the highly conserved catalytic tetrad K-D-K-E in site-specific 2′-O-ribose MTases [10], and this catalytic tetrad was also present in our sequence alignment. Furthermore, a sequence alignment revealed the highly conserved amino acids involved in SAM binding. However, interestingly, the 3D structure of human FTSJ2 showed a different orientation for the first residue of the catalytic tetrad (lysine) compared with RrmJ. This difference may indicate a different A-loop structure of the rRNA substrate or a different catalytic mechanism of the FTSJ2/Mrm2p protein in mammals.

Because *S. cerevisiae* Mrm2p is localized in the mitochondria [12], we hypothesized that hFTSJ2 is a mitochondrial protein. We used immunofluorescence and Western blot analysis to verify that hFTSJ2 was predominantly located in the mitochondria but not in the nucleus or cytoplasm (Figures 3C and 3D). In addition, *E. coli* RrmJ is well known as a heat shock protein. The *rrmJ* mRNA expression increases over 20-fold after heat shock [3], and the *rrmJ* deletion strain fails to adapt to heat shock temperatures [7]. In *S. cerevisiae*, the growth of the mrm2 deletion strain at 37°C is slightly reduced on glucose-containing medium and severely reduced on glycerol-containing medium [12]. Thus, to evaluate the heat shock response of the RrmJ ortholog in mammals, we tested the heat shock response of piglets at 30°C or 35°C. The large intestine, lung and bladder showed an up-regulated expression of *Ftsj2* mRNA at temperatures of 30°C and 35°C, but only the lung tissue demonstrated a simultaneous heat shock response with the up-regulation of *Hsp70.2* mRNA (Figure 4). This finding in the lung may have been caused by the direct exposure of this tissue to the increased temperature through the inhalation of hot air. However, under these heat shock treatments for the piglets, only 5 (small intestine, muscle, lung, kidney, and liver) of the 11 tissues showed an up-regulation tendency of *Hsp70.2* expression, possibly because of the systemic effect of the response of the warm-blooded piglets to the heat shock stress. Furthermore, to eliminate this systemic effect and to confirm the *FTSJ2* mRNA up-regulation in the lung, a human lung adenocarcinoma cell line (A549) was subjected to heat shock for 1 hour and allowed to recover at 37°C. The results of this experiment showed a 1.5-fold increase in the h*FTSJ2* expression at both 42°C and 45°C and then a gradual return to its normal level after the recovery period (Figure 5). Although the exact role of FTSJ2 in the heat shock response in mammals is unknown, these results indicate that FTSJ2 inherited the HSP characteristics of its orthologs in *E. coli* and *S. cerevisiae*. In the previous studies of HSPs, such as HSP70 and HSP90, it has been demonstrated that the heat shock responses are highly conserved during evolution. From *Prokaryota* to *Eukaryota* and *Protozoa* to *Metazoa*, the HSPs represented the universal protein structures and similar physiological functions, and following evolution, the HSPs diverged and translocated into different organelles [1], [2], [38]–[40]. These characteristics are in alignment with the results of the RrmJ phylogenic analysis and the conservation of the heat shock response properties in mammals.

In addition to certain small HSPs (i.e., HSP32, HSP25, and HSP22), most of the HSPs are expressed in all types of tissues [2], [41], and our results showed that *Ftsj2* was expressed in all of the 13 normal piglet tissues (Figure S2). These results indicated that FTSJ2 was not only involved in the heat shock response but also might be necessary for mitochondrial functions under normal condition, according to the mitochondrial localization of FTSJ2. In addition, previous functional studies have shown that the HSPs (i.e., HSP70 and HSP90) are involved in tumorigenesis and the inhibition of apoptosis in cancer cells [40], [42]–[44]. Similarly, the amplification of the genetic locus of *FTSJ2* has been discovered in several NSCLC clinical samples and was considered as a novel oncogenic locus [20]. In our study, the expression of FTSJ2 was also shown in different cancer cells (hepatocarcinoma, lung adenocarcinoma, and rhabdomyosarcoma cells). However, in the human lung adenocarcinoma cell sublines CL1-0 and CL1-5 [45], we found that h*FTSJ2* mRNA was decreased in the more invasive CL1-5 cells compared with the less invasive CL1-0 cells. Moreover, the TE671-h*FTSJ2* cells, which over-expressed the hFTSJ2 protein, showed a decrease in cell migration and invasion (Figure 6). These results indicate that the mitochondrial hFTSJ2 protein exhibits an additional function to suppress cancer cell metastasis. Previous reports have suggested that hFTSJ2 functions in the mitochondria. Thus, it is reasonable that FTSJ2 is required for extensive ATP production through respiration in the mitochondria of proliferating cancer cells [46]–[48]. In contrast, according to recent studies, a mitochondrial complex I and NAD^+^/NADH imbalance enhances the metastasis of breast cancer cell lines [49], and the dynamics of mitochondrial fusion or fission also regulates cell migration and invasion [50], [51]. These results indicate that the invasiveness of cells is affected by the condition and state of their mitochondria.

In conclusion, we characterized FTSJ2 as an ortholog of the *E. coli* 23S rRNA 2′-O-ribose MTase and showed that it functions in the mitochondria. We also provided evidence that FTSJ2 is a novel heat shock protein that is over-expressed after heat shock treatment in both piglet lung and lung adenocarcinoma cells. Surprisingly, FTSJ2 may also be involved in the inhibition of cancer cell migration and invasion by influencing the mitochondrial functions.

### Accession Numbers

The GenBank accession numbers of the protein sequences, which were used in the phylogenetic tree construction and the protein sequences alignment, are labeled in Figure 1 and Figure 2. The protein coding regions of the porcine *Ftsj1* and *Ftsj2* mRNA were first sequenced in this study, and the GenBank accession numbers of the corresponding porcine *Ftsj1* and *Ftsj2* mRNA are EU694401 and EU694400, respectively, and the porcine FTSJ1 and FTSJ2 proteins are ACH57153 and ACH57152, respectively.

## Supporting Information

Figure S1 **Three-dimensional protein structures of** ***E. coli*** **RrmJ, human FTSJ2 and porcine FTSJ2.** (**A**) The protein structure of *E. coli* RrmJ, which was resolved by Bügl *et al.* (2000) (PDB ID: 1EIZ) [7]. (**B**) The protein structure of human FTSJ2, which was resolved by Wu *et al.* (2009) (PDB ID: 2NYU) [36]. (C) The protein structure of porcine FTSJ2, which was predicted using the SWISS-MODEL website with human FTSJ2 as a template. The α-helices and β-strands are shown in green and yellow, respectively. The SAM residues and the K-D-K-E catalytic center are shown in the ball and stick representations, respectively.
(TIF)

Figure S2 **Porcine** ***Ftsj2*** **mRNA expression in porcine tissues.** (**A**) Expression of porcine *Ftsj2* mRNA, as measured by semi-quantitative RT-PCR. Porcine *β-actin* mRNA was used as a loading control. (**B**) Quantification of the porcine *Ftsj2* mRNA expression, which normalized to the *β-actin* mRNA expression. The values are equal to = the means of duplicate experiments.
(TIF)
