# Supplementary figures and images for: FTSJ2, a Heat Shock-Inducible Mitochondrial Protein, Suppresses Cell Invasion and Migration
Source: PLoS One. 2014 Mar 4;9(3):e90818. doi: 10.1371/journal.pone.0090818 (PMC3942483; doi:10.1371/journal.pone.0090818)

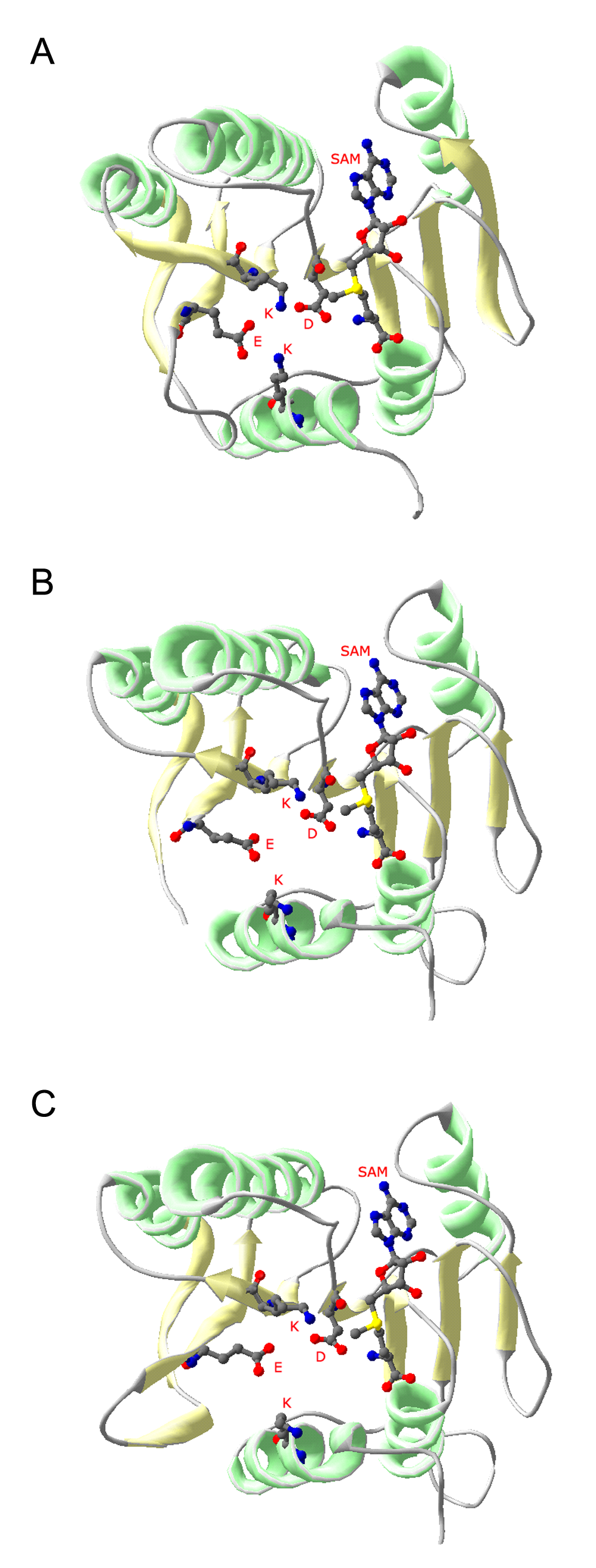

Supplement: Figure S1 — Three-dimensional protein structures of E. coli RrmJ, human FTSJ2 and porcine FTSJ2. (A) The protein structure of E. coli RrmJ, which was resolved by Bügl et al. (2000) (PDB ID: 1EIZ) [7]. (B) The protein structure of human FTSJ2, which was resolved by Wu et al. (2009) (PDB ID: 2NYU) [36]. (C) The protein structure of porcine FTSJ2, which was predicted using the SWISS-MODEL website with human FTSJ2 as a template. The α-helices and β-strands are shown in green and yellow, respectively. The SAM residues and the K-D-K-E catalytic center are shown in the ball and stick representations, respectively. (TIF) [file pone.0090818.s001.tif]

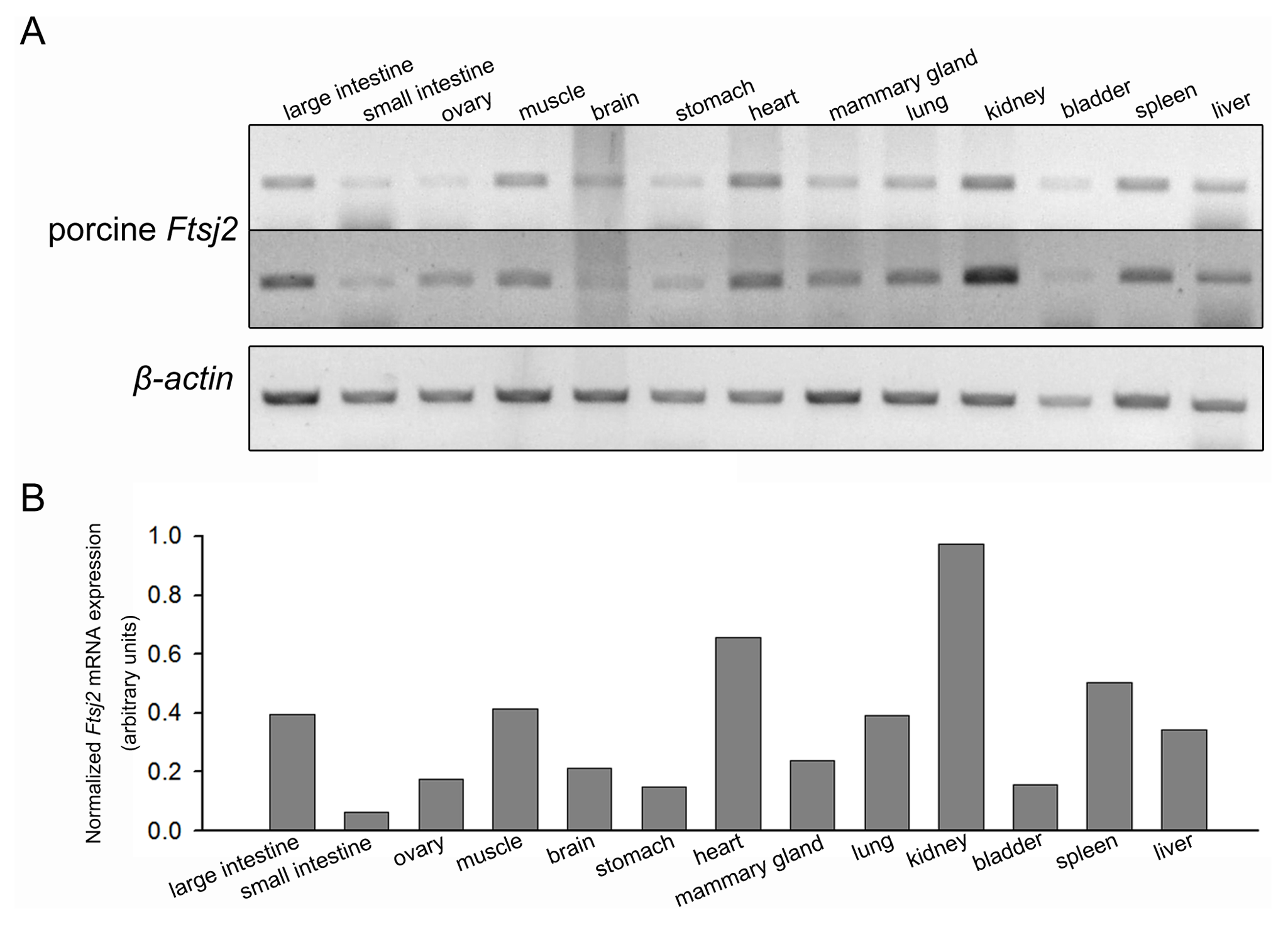

Supplement: Figure S2 — Porcine Ftsj2 mRNA expression in porcine tissues. (A) Expression of porcine Ftsj2 mRNA, as measured by semi-quantitative RT-PCR. Porcine β-actin mRNA was used as a loading control. (B) Quantification of the porcine Ftsj2 mRNA expression, which normalized to the β-actin mRNA expression. The values are equal to = the means of duplicate experiments. (TIF) [file pone.0090818.s002.tif]
